# On the use of haplotype phylogeny to detect disease susceptibility loci

**DOI:** 10.1186/1471-2156-6-24

**Published:** 2005-05-18

**Authors:** Claire Bardel, Vincent Danjean, Jean-Pierre Hugot, Pierre Darlu, Emmanuelle Génin

**Affiliations:** 1 Unité de recherche en Génétique Épidémiologique et structure des populations humaines, INSERM U535, Villejuif, France; 2 Laboratoire Bordelais de Recherche en Informatique, UMR 5800, Bordeaux, France; 3 Programme Avenir, INSERM U458, hôpital Robert Debré, AP-HP, Paris, France; 4 Fondation Jean Dausset, Paris, France

## Abstract

**Background:**

The cladistic approach proposed by Templeton has been presented as promising for the study of the genetic factors involved in common diseases. This approach allows the joint study of multiple markers within a gene by considering haplotypes and grouping them in nested clades. The idea is to search for clades with an excess of cases as compared to the whole sample and to identify the mutations defining these clades as potential candidate disease susceptibility sites. However, the performance of this approach for the study of the genetic factors involved in complex diseases has never been studied.

**Results:**

In this paper, we propose a new method to perform such a cladistic analysis and we estimate its power through simulations. We show that under models where the susceptibility to the disease is caused by a single genetic variant, the cladistic test is neither really more powerful to detect an association nor really more efficient to localize the susceptibility site than an individual SNP testing. However, when two interacting sites are responsible for the disease, the cladistic analysis greatly improves the probability to find the two susceptibility sites. The impact of the linkage disequilibrium and of the tree characteristics on the efficiency of the cladistic analysis are also discussed. An application on a real data set concerning the CARD15 gene and Crohn disease shows that the method can successfully identify the three variant sites that are involved in the disease susceptibility.

**Conclusion:**

The use of phylogenies to group haplotypes is especially interesting to pinpoint the sites that are likely to be involved in disease susceptibility among the different markers identified within a gene.

## Background

With the development of molecular techniques to identify genetic polymorphisms, numerous markers and in particular Single Nucleotide Polymorphisms (SNPs) [1,2] are now available within and between genes for establishing their possible role in disease susceptibility. One recurrent question in the literature is the way these markers could be used to test for association with complex diseases [3,4]. Should one focus on one marker at a time and simply compare allele or genotype distributions for the studied markers in cases and controls or should one consider haplotypes formed by several linked markers? Haplotypic methods have been suggested to be more powerful at detecting the role of a given genomic region in disease susceptibility [4-7]. Indeed, disease susceptibility may be due to the combined effects of variants at different markers. Different methods have been proposed to test for association between markers located on the same reconstructed haplotypes and disease susceptibility [4,7-10]. In these tests, the number of haplotypes being large, the degrees of freedom to compare cases and controls are also large, decreasing the power to detect the association. Moreover, as some haplotypes would only be carried by one or two individuals, there could be statistical problems owing to small sample sizes making difficult the evaluation of their possible effect on the susceptibility to the disease.

A solution to reduce these problems is to group haplotypes in order to decrease the degrees of freedom and to increase the number of individuals in the different haplotype groups. The cladistic method as described by Templeton et al. [11] is a method to carry out such groupings of haplotypes and to determine which haplotype or group of haplotypes is likely to be responsible for the phenotypic variation observed among the population. It consists in using parsimony methods to build a phylogeny of the haplotypes, and to group haplotypes according to the clades defined by the phylogenetic tree. When applied to a case/control data set, the proportions of cases and controls in different clades are compared. If a clade shows a significantly larger number of cases than the others, one will conclude, first, that there is an association between the disease and one haplotype or a group of haplotypes belonging to the clade, and, second, that the mutations defining the clade containing an excess of cases are good candidates to be functional gene polymorphisms involved in the disease susceptibility.

The cladistic analysis relies for a large part on the reconstruction of an accurate phylogenetic tree, but recombination is known to bias phylogenetic reconstruction processes [12]. The recent discovery of the haplotype block structure of the human genome [13] now provides an interesting framework for cladistic methods. Indeed, haplotype blocks define regions in which the recombination rate is quite low. Moreover, once a block is found to be associated with a disease, it will not be suitable to use classical methods based on recombination to fine map susceptibility loci. Haplotype phylogenies being based on the history of mutations may then help to identify disease susceptibility loci.

The cladistic method was first described in a series of five articles [11,14-17] and applied in different case-control studies focusing either on quantitative or qualitative data. For example, Haviland et al. used a cladistic analysis to study the association between the apolipoprotein AI-CIII-AIV and plasma lipid, lipoprotein and apolipoprotein levels [18]. Keavney et al. [19] used cladograms to localize the sites that significantly influence Angiotensin-I Converting Enzyme (ACE). Zhu et al. [20] worked on quantitative data simulated for the twelfth Genetic Analysis Workshop (GAW). Using regression analysis and analysis of variance (ANOVA), they were able to find a short region containing the simulated mutation. Wang et al. [21] used a nested linear model to analyze the association between *ApoA4 *haplotypes and plasma lipid level in the cladistic framework. Among the studies that consider discrete data is the one by Kittles et al. [22] who tested the association between haplotype variations on the Y chromosome and alcohol dependence and related personality traits. Lobos and Todd applied the same cladistic method to four RFLPs in the tyroxine hydroxylase gene [23], and to five SNPs in the dopamine receptor D2 gene [24]. Darlu and Génin [25] applied the method to the GAW 12 simulated data and were able to identify some of the disease susceptibility sites. For a more detailed review on the subject, see [26].

More recently Seltman et al. [27] developed a method that can be applied on family-based data. They proposed a test, the ET-TDT, that combines the transmission disequilibrium test (TDT) and the use of unrooted phylogenetic trees to group haplotypes. They further extended their method to the analysis of qualitative and quantitative data from case-parent trios or population-based studies [28]. Finally, an alternative method where phylogenetic trees are reconstructed by a distance method was proposed by Durrant et al. [29] and implemented in the software CLADHC.

From these different examples, cladistic methods appear to be innovative methods to detect an association between a candidate gene and a disease and to localize disease susceptibility sites. However, the validity and the power of these methods to detect association have only been tested in a very limited number of situations (fixed pattern of linkage disequilibrium, fixed phylogenetic tree) [27,29]. In this paper, we develop a new cladistic test (CT), based on the analysis of a rooted tree, which allows to make hypotheses about functional polymorphisms that are involved in the susceptibility to the disease. Through simulations on ten different tree topologies, we evaluate the conditions in which this cladistic method is efficient and compare it to other methods. Finally, we apply this method to a real data set concerning Crohn disease and the CARD15 gene [30].

## Results

### Association test

As detailed in the method section, four different tests are performed: a single locus allele-based test referred to as SbST (Site by Site Test), an haplotype-based test referred to as HT (Haplotypic Test), a test based on haplotype phylogenies referred to as CT (Cladistic Test) and a test based on the clustering of haplotypes described by Durrant et al. [29] and referred to as CLADHC. To compare their respective power to detect an association, simulations were performed under three disease models involving one (model 1) or two (model 2 and model 3) susceptibility sites for ten different tree topologies.

Results for model 1 are presented in Table 1 and 2. When the susceptibility site is included in the studied SNPs (Table 1), SbST is the most powerful test for all trees but one (tree 8) (between 99.1% and 99.5% depending on the tree, 99.35% on average), while the other tests have a weaker power (92.9%, 93.7%, and 91.2%, on average, for CT, HT, and CLADHC respectively). CT and CLADHC have almost the same average type I error, slightly but significantly lower than the type I error of HT and SbST. In these simulations, as the type I errors cannot be fixed a priori, powers may be difficult to compare. However, if the type I errors of CT and CLADHC were increased, their power would also be increased and, consequently, the difference in power with HT would be even more pronounced and the difference in power with SbST would be more reduced. In the trees showing comparable type one error (Trees 1, 5, 7), the power of SbST is clearly higher than the power of CT. When the susceptibility site is removed (Table 2), the performances of the four tests are, on average, similar (about 89%), CT being the most powerful test for four trees, SbST for four other trees, HT and CLADHC for one tree each. However, the type I errors being slightly different, by adjusting them one expects that CT and CLADHC could be on average, slightly more powerful than HT and SbST.

**Table 1:** Power to detect an association, one susceptibility site simulated and included in the analysis

Tree	CT	CLADHC	HT	SbST
1	96.4 *(3.3)*	88.9 *(3.3)*	91.1 *(5.1)*	99.3*(3.4)*
2	99.0 *(4.4)*	95.7 *(4.1)*	91.3 *(6.0)*	99.5*(5.3)*
3	86.0 *(2.5)*	91.1 *(4.7)*	91.2 *(4.6)*	99.3*(4.4)*
4	96.3 *(3.8)*	95.6 *(2.9)*	91.5 *(4.2)*	99.3*(4.6)*
5	88.8 *(4.1)*	95.3 *(3.3)*	90.4 *(4.7)*	99.1*(4.6)*
6	84.5 *(3.5)*	88.8 *(2.7)*	90.7 *(4.4)*	99.5*(4.4)*
7	97.4 *(4.0)*	96.3 *(3.7)*	91.9 *(4.8)*	99.2*(4.3)*
8	99.7* (3.2)*	98.5 *(2.4)*	91.2 *(4.9)*	99.4 *(4.7)*
9	88.1 *(3.5)*	94.0 *(4.4)*	91.3 *(4.7)*	99.4*(4.3)*
10	92.5 *(2.6)*	93.0 *(2.9)*	91.3 *(4.7)*	99.5*(3.6)*

Average	92.9 *(3.5)*	93.7 *(3.4)*	91.2 *(4.8)*	99.35*(4.4)*
Std dev.^a^	5.63 *(0.62)*	3.24 *(0.76)*	0.41 *(0.49)*	0.13 *(0.54)*

Sample size: 100 cases and 100 controls, 1000 replicates, penetrance vector: [0.03;0.06;0.3], frequency of the susceptibility allele: 0.205 ± 0.005. The corresponding type I errors are indicated in italics within brackets. For each tree, the method giving the best result is underlined. CT: Cladistic Test, CLADHC: test developed by Durrant et al. [29], HT: Haplotypic Test, SbST: Site by Site Test.

^a ^Standard deviation

**Table 2:** Power to detect an association, one susceptibility site simulated and removed from the analysis

Tree	CT	CLADHC	HT	SbST
1	94.8* (3.8)*	89.1 *(3.5)*	91.2 *(5.6)*	89.5 *(3.9)*
2	94.0 *(3.8)*	92.7 *(4.1)*	87.6 *(5.6)*	98.5* (5.3)*
3	79.3 *(3.2)*	88.9 *(4.7)*	86.2 *(4.7)*	94.2* (4.1)*
4	88.7 *(2.3)*	85.7 *(3.1)*	87.8 *(4.6)*	93.6* (4.4)*
5	85.6 *(4.3)*	94.4* (4.1)*	90.5 *(4.5)*	94.0 *(4.1)*
6	78.6 *(3.0)*	81.6 *(3.3)*	88.0* (4.2)*	67.0 *(4.2)*
7	92.4 *(3.8)*	90.5 *(3.7)*	87.8 *(5.2)*	92.7* (4.0)*
8	99.7* (3.0)*	98.6 *(2.8)*	91.2 *(4.7)*	99.6 *(4.5)*
9	91.9* (3.7)*	89.2 *(4.5)*	91.2 *(4.8)*	89.1 *(4.1)*
10	88.8* (2.9)*	82.8 *(3.0)*	81.3 *(4.1)*	79.1 *(3.5)*

Average	89,38 *(3.4)*	89.35 *(3.7)*	88,28 *(4.8)*	89,73* (4.2)*
Std dev.^a^	6.71 *(0.6)*	5.2 *(0.6)*	3.06 *(0.5)*	9.81 *(0.5)*

Sample size: 100 cases and 100 controls, 1000 replicates, penetrance vector: [0.03;0.06;0.3], frequency of the susceptibility allele: 0.205 ± 0.005. The corresponding type I errors are indicated in italics within brackets. For each tree, the method giving the best result is underlined. CT: Cladistic Test, CLADHC: test developed by Durrant et al. [29], HT: Haplotypic Test, SbST: Site by Site Test.

^a ^Standard deviation

As expected, the power is higher when the susceptibility site is included than when it is removed. In this latter case, the power depends on the degree of linkage disequilibrium (LD) between the susceptibility site and the other sites as expressed by the maximum value of LD, referred to as *LD*_*max*_. Moreover, since the pattern of LD can be different from one tree to another, one expects more differences in power between trees when the susceptibility site is removed. Indeed the standard deviation of the power is larger in this case. This standard deviation is also particularly large for SbST (*σ *= 9.81). This test, being an allele-based test, is more influenced by LD than the three others tests that are haplotype-based tests, as proved by the high positive correlation observed between the power of SbST to detect association and the *LD*_*max *_(Spearman rank correlation ρ equals 0.851, p-value < 0.01). For CT and CLADHC, Spearman rank correlations are not significant (p > 0.1) confirming that LD has a lesser impact on these two tests. Therefore, the high standard deviations observed for these two tests are probably more related to the phylogeny of haplotypes than to the linkage disequilibrium.

The results for model 2 and model 3, differing by the values of the penetrance vector, are presented in Table 3 and in Table 4 respectively. There is no real difference in power between the four tests for model 2 (about 97%). For model 3, the power of the four tests is approximately half the power for model 2 owing to the reduced value of the penetrance for the genotype associated to the highest risk in this model. In this case, SbST and CT are, on average, the most and the less powerful test respectively

**Table 3:** Power to detect an association for two susceptibility sites simulated under model 2

		Model 2		
Tree	CT	CLADHC	HT	SbST

1	97.8 *(3.7)*	99.3* (3.8)*	97.7 *(5.0)*	99.1 *(6.9)*
2	95.1 *(3.6)*	97.7 *(2.9)*	97.9* (5.6)*	95.8 *(4.3)*
3	94.2 *(4.9)*	99.4* (4.9)*	97.9 *(5.1)*	98.6 *(4.9)*
4	97.6 *(4.6)*	97.8 *(4.6)*	97.8 *(5.5)*	98.6* (4.6)*
5	98.5 *(5.3)*	99.5* (4.5)*	98.9 *(5.7)*	98.2 *(7.4)*
6	98.9* (4.4)*	97.9 *(3.9)*	98.5 *(5.7)*	96.9 *(6.6)*
7	93.0 *(5.1)*	93.5 *(4.6)*	96.9* (5.6)*	95.5 *(5.2)*
8	95.2 *(6.1)*	99.0* (5.3)*	98.2 *(6.2)*	95.4 *(4.2)*
9	96.7 *(3.6)*	97.3 *(3.1)*	97.6 *(4.1)*	97.7* (4.0)*
10	99.9* (4.5)*	99.5 *(4.0)*	98.2 *(60.)*	99.8 *(5.8)*

Average	96.7 *(4.6)*	98.06* (4.2)*	97.96 *(5.45)*	97.56 *(5.4)*
Std dev.^a^	2.24 *(0.8)*	1.83 *(0.8)*	0.54 *(0.6)*	1.58 *(1.2)*

Sample size: 200 cases and 200 controls, 1000 replicates, penetrance: 0.9 for the homozygotes carrying the two susceptibility alleles, 0.3 for the other genotypes. The corresponding type I errors are indicated in italics within brackets. For each tree, the method giving the best result is underlined. CT: Cladistic Test, CLADHC: test developed by Durrant et al. [29], HT: Haplotypic Test, SbST: Site by Site Test

^a ^Standard deviation

**Table 4:** Power to detect an association for two susceptibility sites simulated under model 3

		Model 3		
Tree	CT	CLADHC	HT	SbST

1	40.7 *(4.7)*	54.0* (4.5)*	42.0 *(5.9)*	47.5 *(4.7)*
2	31.7 *(3.4)*	32.7 *(3.9)*	43.0 *(5.9)*	45.8* (5.3)*
3	26.8 *(4.2)*	47.9* (4.4)*	40.7 *(6.4)*	45.2 *(3.5)*
4	34.8 *(4.8)*	39.2 *(4.7)*	38.9 *(7.0)*	47.0* (4.4)*
5	41.5 *(5.6)*	49.7* (4.7)*	44.2 *(6.6)*	37.2 *(4.6)*
6	42.0 *(3.6)*	34.8 *(4.7)*	42.4* (5.6)*	41.3 *(5.3)*
7	28.6 *(5.2)*	32.1 *(4.7)*	41.6* (6.4)*	39.9 *(4.3)*
8	33.2 *(4.3)*	47.2* (4.0)*	42.9 *(6.1)*	37.7 *(4.0)*
9	33.5 *(4.4)*	39.1 *(4.1)*	41.2 *(7.0)*	48.5* (5.3)*
10	65.6* (6.6)*	48.1 *(4.1)*	42.1 *(7.8)*	56.9 *(4.2)*

Average	37.84 *(4.7)*	42.48 *(4.4)*	41.9 *(6.5)*	44.7* (4.6)*
Std dev.^a^	11.07 *(0.9)*	7.84 *(0.3)*	1.45 *(0.7)*	5.94 *(0.6)*

Sample size: 200 cases and 200 controls, 1000 replicates, penetrance: 0.6 for the homozygotes carrying the two susceptibility alleles, 0.3 for the other genotypes. The corresponding type I errors are indicated in italics within brackets. For each tree, the method giving the best result is underlined. CT: Cladistic Test, CLADHC: test developed by Durrant et al. [29], HT: Haplotypic Test, SbST: Site by Site Test

^a ^Standard deviation

### Localization of the susceptibility site(s)

**Figure 1:**
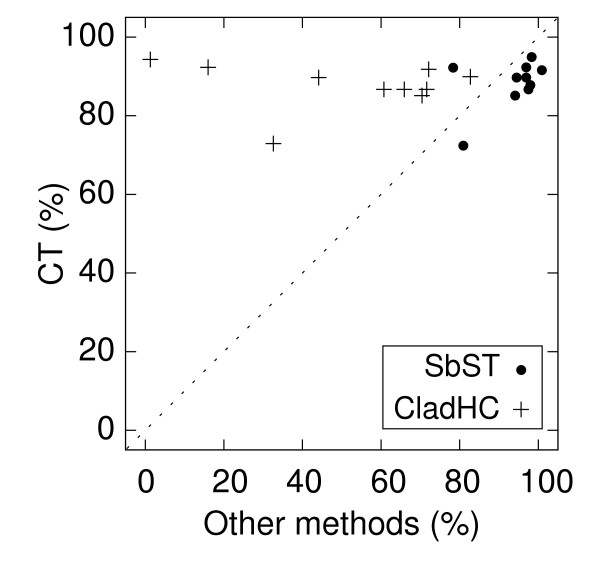
**Comparison of the efficiency to localize the susceptibility site between CT, and SbST or CLADHC**. The efficiency to localize the susceptibility site is measured by the percentage of replicates in which the simulated susceptibility site is either uniquely detected or detected among other putative sites. black circles: comparison of CT and SbST, crosses: comparison of CT and CLADHC. Each symbol corresponds to one of the ten tree topologies.

**Figure 2:**
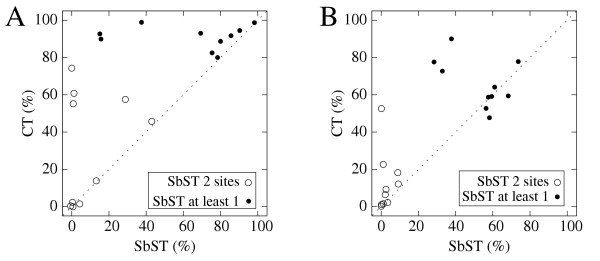
**Comparison of the efficiency to localize the two susceptibility sites between CT and SbST**. The efficiency to localize the two susceptibility sites is measured by the percentage of replicates in which the two sites are detected (black circles) or at least one of the two sites (white circles) Each symbol corresponds to one of the ten tree topologies. (A): penetrance 0.9 for homozygotes carrying the two susceptibility alleles and 0.3 for all other genotypes; (B): penetrance 0.6 for homozygotes carrying the two susceptibility alleles and 0.3 for all other genotypes.

The efficiency of SbST, CT and CLADHC to localize the susceptibility site(s) when it is included among the SNPs is presented in Figure 1 and 2. The efficiency of CT is plotted against the efficiency of SbST or CLADHC. To correctly interpret these figures, it has to be noted that the criteria used to compare the methods are not strictly identical. Indeed, the identified SNP is always unambiguously detected by the SbST method, since this is the site giving the most significant chi-square and two chi-squares are never exactly identical. On the other hand, the susceptibility site may not be the only one detected by CT because two or more sites could have the same values of the criteria (*V*_*i*_, see the Methods section). However, in our simulations, the susceptibility site is the only one detected in from 80% (for 1 tree) to 100% (for 5 trees) of the replicates. When the susceptibility site is not found alone, it is almost always found with only one other site, rarely with two other sites (<0.5% of the replicates).

For model 1 (Figure 1), CT is slightly less efficient than SbST (for 9 trees out of 10) and more efficient than CLADHC (for all 10 trees). For SbST, we note a high negative correlation between the efficiency to localize the susceptibility site and the *LD*_*max *_between the susceptibility site and the other sites (Spearman rank correlation *ρ *equal -0.88 with a p-value of 0.008). It shows that the presence of a site in relatively high LD with the susceptibility site disturbs the localization process, increasing the uncertainty of the susceptibility site's identification. Interestingly, there is no such statistically significant correlation with CT and CLADHC (p > 0.08), due to the fact that these last two tests use both the information on the whole haplotypes and on their history through the tree reconstruction. The presence of sites in high LD with the susceptibility site does not prevent these two methods from localizing correctly the susceptibility site.

For model 2 and model 3, (Figure 2A and 2B respectively), CT is more or at least as efficient as SbST to localize the two sites, whatever the penetrance model. For the detection of at least one of the two sites, we can observe that, whatever the tree, CT is always more efficient than SbST under model 2, whereas this is only true for 6 out of the 10 trees under model 3.

### Application to CARD15/NOD2 polymorphisms and Crohn disease

The reconstruction of the phylogenetic tree of the 33 haplotypes leads to a very high number of equally parsimonious trees. One thousand of them were retained and analyzed. In table 5, for the two rooting methods mfH and consH, the SNPs are ranked from the least to the most significant (based on the *V*_*i *_parameter, see the Methods section). Association is detected with CT (*p*_*mfH *_= 2 × 10^-5^, *p*_*consH *_= 3 × 10^-5^) and SNP 8 is selected as potential susceptibility site. If haplotypes carrying the susceptibility allele at SNP 8 are removed, an association can still be detected (*p*_*mfH *_= 4 × 10^-5^, *p*_*consH *_= 3 × 10^-5^) and SNP 12 is identified as potential susceptibility site. When removing haplotypes with the susceptibility allele at SNP 12, there is still a remaining association (*p*_*mfH *_= 8 × 10^-5^, *p*_*consH *_= 3 × 10^-5^) and depending on the rooting method either SNP 13 (with consH) or SNP 9 (with mfH) are retained. When haplotypes with the susceptibility allele at SNP 13 are removed, the association test is not significant (*p*_*mfH *_= 2.9 × 10^-1^, *p*_*consH *_= 1.1 × 10^-1^). When haplotypes with the susceptibility allele at SNP 9 are removed, the association test is significant (*p*_*mfH *_= 2 × 10^-5^, *p*_*consH *_= 5 × 10^-5^) and the identified site is SNP 13 for both rooting method. In the end, the combination of SNP 8, SNP 12 and SNP 13 is the most parsimonious combination that can explain the susceptibility to the disease. Moreover, we can note that these 3 SNPs are those showing the highest *V*_*i *_on the whole data set. So, we finally have identified three SNPs involved in the susceptibility to Crohn's disease.

**Table 5:** Cladistic analysis of Crohn's data 1000 equiparsimonious trees are studied. SNPs are ranked from the least to the most significant. In bold, the presumed susceptibility sites previously identified by Hugot et al. [30].

	least significant				-------->	most significant		
mfH^a^	SNP 5	SNP 9	SNP 6	SNP 7		**SNP 13**	**SNP 12**	**SNP8**
consH^b^	SNP 9	SNP 5	SNP 6	SNP 7		**SNP 13**	**SNP 12**	**SNP8**

^a ^Rooting on the most frequent haplotype

^b ^Rooting on a consensus sequence

## Discussion

In this paper, we have presented a method to perform phylogeny-based nested haplotype analysis of case/control data and introduced the cladistic test (CT). We have shown by simulations that the CT test is approximately as powerful to detect an association as a test that compares haplotype distributions in cases and controls with no prior grouping. However, a major advantage of CT towards other haplotypic tests is the possibility of making inferences on potential susceptibility sites. Indeed the method of parsimony used to build the phylogeny allows the reconstruction of character state changes on the tree and thus the identification of the most likely susceptibility sites. Finding the polymorphisms responsible for the susceptibility to a disease is an important issue in genetic epidemiology and in the past few years, different methods have been proposed that use linkage disequilibrium to fine-map candidate genes and to localize disease mutations ([31,32] and [33] for example) These methods are based on the fact that a disease variant arises on a particular haplotype, the haplotype and the disease thus being initially associated. Then, over time, this association is broken by recombination and only a small region of the ancestral haplotype is preserved around the disease variant. In such models, recombination is the event of interest. Other parameters such as mutations can be integrated in the models, but they are considered as nuisance parameters. The cladistic test developed here is different in the sense that it is based on the reconstruction of the evolutionary history of the mutations that occur to form the haplotypes. Contrary to the other fine-mapping methods, the event of interest is mutation, and recombination is a nuisance parameter. Through the simulation study, we have shown that this test is a powerful tool to suggest etiological SNPs. This is also illustrated here on Crohn disease since the cladistic test was able to correctly identify the 3 sites within the CARD15 gene that are already reported to be involved in the disease susceptibility [30].

The cladistic test requires the building of a phylogeny of the different haplotypes present in the sample. Different methods are available to build haplotype phylogenies: distance methods, parsimony methods and maximum likelihood (ML) methods. A parsimony method was chosen here because it allows the reconstruction of apomorphies that can be used to define potential sites involved in the disease susceptibility and because it runs faster than ML methods, thus allowing a simulation study. We have compared CT with another clustering method, CLADHC, a distance-based method that also differs from CT in the statistical analysis (CLADHC performs regression analysis, CT is based on chi-square tests). We have shown that the power of these two tests to detect association is quite similar, CT being slightly more powerful when one site is simulated, and CLADHC when two sites are simulated. For the localization of the susceptibility site, the efficiency of the two methods is difficult to compare because CT directly localizes the site on short sequences while CLADHC localizes a window containing the susceptibility site on longer sequences. The two test should thus be used in different situations. Nevertheless CT appears to be more efficient in our simulation conditions

In the phylogeny reconstruction, one is faced to different problems. The first one is the choice of the ancestral sequence to root the phylogeny. With real data, the ancestral sequence is usually not known, unless at least two outgroups can be postulated (in the parsimony context). In the absence of ancestral sequences, hypotheses have to be made, and they are numerous, like taking the most frequent haplotype, but this haplotype could be different from one population to an other or from one sampling to another, and this most frequent haplotype may not have a significantly different frequency from the next most frequents. Or one can take the consensus haplotype, but this sequence may not be observed in the sample, or not be among the most frequent sequences. In this study, our strategy was to consider the uncertainty of the ancestral sequence as a nuisance parameter, since our goal was not primarily to focus on this problem and because we must be in the same situation for all the performed simulations. Extensive further simulations will be needed to assess the properties of the different rooting methods. However, several ancestral hypotheses should be considered, discussed, and tested on real data, as we have done for the Crohn data. Second, the presence of recombinations would restrict the use of phylogenetic reconstruction. Indeed, phylogenetic reconstruction is known to be biased by recombinations [12] in a manner depending on how and when the recombination events occur in the tree. This context led us to simulate the history of mutations along the haplotypes without introducing recombinations between haplotypes. However, because in our method the reconstructed tree has not to represent the exact history of the haplotypes, but only gives a way to cluster them in an appropriate way, we believe that our method can tolerate some low degrees of recombination, as it is probably the case for the Crohn data. Further simulations would however be required to better assess this point. Third, the choice of the way characters are optimized on the tree is another important point in cladistic analysis. Two classical options can be used to optimize the character state changes along the tree: deltran, (delayed transformation) that favors convergences or acctran(accelerated transformation) that favors reversions. In this paper, only the results performed with the deltran option are presented. Simulations (one susceptibility site) have also been performed with the acctran option. For the association detection, the results are very similar to those obtained with deltran showing that this parameter does not influence the power of the CT method to detect association in our simulation conditions. For the localization of the susceptibility site, the results are different. The method is more efficient with the deltran option than with the acctran option for eight trees out of ten, it is less efficient for one tree and the results are equivalent for the last tree. It is partly due to to the uncertainties of the character *S *and to the frequency of the disease susceptibility allele. When the frequency of the susceptibility allele is low, as in our simulations, and when *S *is coded "?" in some nodes of the tree, one expects the change of *S *from 0 to 1 to occur later in the tree, and the delayed option (deltran) will then be more appropriate. Conversely, when the frequency of the disease is large, the change of *S *from 0 to 1 has more chances to occur earlier in the tree since the *S *mutation can then include more cases, and the accelerated option (acctran) will be more appropriate. However, when analyzing a real data set, it is advisable to compare the results obtained with both options.

The variability of the results obtained with the cladistic method is due for a minor part to the LD between the susceptibility site(s) and the other sites and for the major part to parameters specific to the tree. Among these parameters, the position of the mutation in the tree (near the root or the leaves, sample size of the clades near the mutations...) has already been reported to have an influence on the power of cladistic methods to detect association [27]. Apart from the position of the mutation, the presence of sites that co-mutate with the susceptibility site, the degree of multiple mutations of the susceptibility site in the tree, and the existence of one or more reversions for the susceptibility site influence the power of the cladistic test to detect an association, and they have an even greater impact on the efficiency of the cladistic test to localize the susceptibility site. As all these factors are not independent, the evaluation of their own effects remains problematic.

The four methods compared here (CT, HT, SbST and CLADHC) suppose that haplotypic data are available, which is not usually the case. The most likely haplotypes for an individual have to be reconstructed using numerical methods (for a review of these methods, see [34,35]). The availability of familial data may help to determine the phase of the markers and then, to obtain the most likely haplotypes by using the genetic information of the two parents. However, even when these familial data are not available, the numerical methods have been shown to be very efficient to reconstruct haplotypes, except for rare haplotypes that may either be missed or falsely created. In this work, since we choose to focus on the comparison between several methods, the problem of haplotype inference is ignored. Moreover, as the four studied methods are all based on already known or inferred haplotypes carried by the individuals, the uncertainty due to the haplotype inference should not play differently from one method to another. Further extension of the CT method will take this uncertainty into account by using, for example, likelihood-based tests.

## Conclusion

In conclusion, as proved by our simulations and the Crohn example, the use of phylogenies to group haplotypes by clades and the nested analysis of case-control data on the basis of these clades turns out to be an interesting and complementary strategy for the genetic study of multifactorial diseases, especially to pinpoint the sites that are likely to be involved in disease susceptibility among the different markers identified within a gene.

## Methods

### Principle of the method

The material on which the method is based is a sample of haplotypes composed of a combination of SNPs, each haplotype being labeled either as control or as affected depending on the phenotype of the individual. After building the phylogenetic tree of these different haplotypes, a series of nested homogeneity tests are performed to detect differences in the distribution of cases and controls in the different clades. Once an association is detected, a new character is defined according to the proportion of cases carrying an haplotype. Then, it is optimized on the tree and the sites that significantly co-mutate with this new character are putative susceptibility sites for the disease.

### Simulation study

To test the power of this method to detect an association and its efficiency to precisely localize the susceptibility site, we performed the following steps : i) simulation of the haplotypes and choice of the susceptibility site(s) (one or two susceptibility sites are considered) ; ii) attribution of a status (case or control) to these haplotypes taking into account the genetic model of the disease; iii) reconstruction of the phylogeny of haplotypes and inference of the character state changes along the branches of the tree; iv) association test: nested analysis of the case/control ratio in the clades; v) localization of the susceptibility site using the equiparsimonious (ie, all the trees that have the minimum number of character state changes) ; vi) estimation, by simulations, of the power to detect the association between haplotypes and the disease and comparison with other methods; vii) estimation of the efficiency to localize susceptibility sites and comparison with other methods.

#### Haplotype simulation

24 different haplotypes are simulated using the TREEVOLVE software (version 1.32) [36]. An ancestral haplotype sequence of 20 sites is randomly produced, a guide tree is generated using the coalescent model without recombination and the evolution of this ancestral sequence along the tree is simulated to obtain a sample of 24 different haplotypes. Since TREEVOLVE can only simulate the evolution of DNA sequences (4 nucleotides) and not the evolution of bi-allelic markers, we set the frequencies for bases *A *and *T *to very low values (*A *= *T *= 0.001 and *G *= *C *= 0.498) in order to obtain an ancestral sequence composed only of *G *and *C*, as an equivalent of SNPs coded by 0/1 character states. The mutation rate is set to 2 × 10^-6^, and the substitution rates are chosen so that only G-C transversions could occur: *A *- *C *= 0.001, *A *- *G *= 0.001, *A *- *T *= 0.001, *C *- *T *= 0.001, *G *- *T *= 0.001, *G *- *C *= 0.995. No heterogeneity in the substitution rate among sites is specified. The population size was set to 100,000 and its growth was stationary with no subdivision. Once the haplotypes are obtained by simulation, the susceptibility sites are chosen among the 20 sites. For the simulations with only one susceptibility site, the program picks up the first site in the sequence having a minor allele frequency (*p*) in the sample of haplotypes within the range *F *<*p *<*F *+ 0.01 where *F *is a predefined allele frequency (in our simulations, *F *= 0.20). For two simulated sites, the program picks up the first two simulated sites verifying a user-defined constraint. In our simulations, the two sites are chosen such that *f *(*A*_1_*B*_1_) = *f *(*A*_1_*B*_2_) = *f *(A_2_B_1_) = *f *(*A*_2_*B*_2_) = 0.25, *A*_1 _and *A*_2 _being the two alleles at the first site and *B*_1 _and *B*_2_, the two alleles at the second site. When no site is found satisfying these conditions, a new set of haplotypes is simulated until the required conditions are obtained.

This entire procedure is repeated ten times to generate ten different data sets. The pairwise linkage disequilibrium (*r*^2 ^measure, mean values for the 1000 replicates) and the tree topologies corresponding to these ten data sets are provided as Additional file 1 and 2.

#### Attribution of disease status

To generate the genotypes of each individual, pairs of haplotypes are sampled at random with replacement and the disease status (case or control) is determined by his genotype at the susceptibility site(s) and by the penetrances. In our simulations, three disease models are studied, involving either one susceptibility site (model 1) or two interacting susceptibility sites (model 2 and model 3). The first model is the disease model described in Bourgain et al. [37] that roughly corresponds to the effect of *ApoE4 *in Alzheimer's disease [38]. We set the penetrance of the three genotypes *DD*, *Dd *and *dd *to respectively *P*_*DD *_= 0.03, *P*_*Dd *_= 0.06, *P*_*dd *_= 0.30, *d *being the disease susceptibility allele. For model 2 and model 3, an at-risk haplotype formed by the combination of the alleles *A*_2 _at the first locus and *B*_2 _at the second locus is defined. The penetrance associated to the genotype *A*_2_*B*_2_*A*_2_*B*_2 _is set to 0.9 for model 2 and to 0.6 for model 3. All the other penetrances are set to 0.3 for both models.

The sampling process is carried on until *N *cases and *N *controls are obtained (*N *= 100 or 200 in our simulations, depending on the number of simulated susceptibility sites). This constitutes a replicate. As the haplotypes are sampled randomly from the set of 24 different haplotype, they are almost equi-frequent in the sample of cases and controls, and their frequencies are close to .

#### Phylogeny of haplotypes

All the different haplotypes are used to build a phylogenetic tree by a parsimony method which consists in choosing the tree that minimizes the number of mutation events [39]. The PAUP software (version 4.0b10 [40]) is used to reconstruct the tree. A heuristic search is performed, using stepwise addition and TBR (tree bisection and reconnection). All sites are equally weighted, and changes from allele 0 to allele 1 and from allele 1 to allele 0 are equally allowed for all sites, including the susceptibility site (unordered option). Among the list of all parsimonious trees, only the first one obtained is used for the association test, for computation time reasons and because we found that identical results are obtained for more than 98% of the equiparsimonious trees (data not shown). For the localization of susceptibility sites, all the equiparsimonious trees (or up to 100 different trees if there are too many equiparsimonious trees) are analyzed. The trees are rooted using the real ancestor obtained with TREEVOLVE. The character states are inferred along the branches by using the classical option deltran (delayed transformation), but acctran (accelerated transformation) was also investigated.

#### Association test

**Figure 3:**
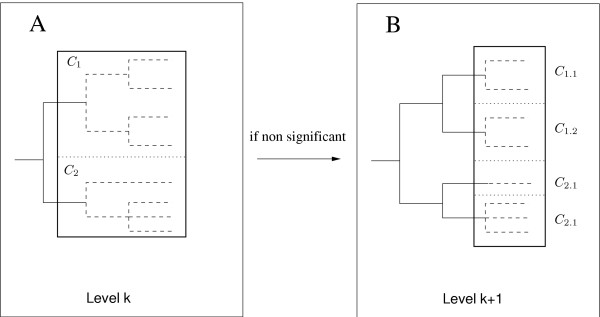
**Description of the nested clade analysis**. (A) shows the homogeneity test performed at level k (between clades *C*_1 _and *C*_2_). If it is not significant (B), a test will be performed at the following level (k+1), between all the sub-clades descending from clades *C*_1 _and *C*_2_, i.e between clades *C*_1.1_, *C*_1.2_, *C*_2.1 _and *C*_2.2 _(3 degree of freedom). If it is significant the analysis ends because an association is detected.

Starting from the root of the tree, series of nested homogeneity tests comparing the number of cases and controls in different clades are performed. The principle of the method is explained in Figure 3. Briefly, at each level of the tree, homogeneity in the distribution of cases and controls is tested among all the *n *clades defined at this level. If the test is significant, an association is detected and the analysis ends. If the test is not significant, one homogeneity test is performed between all the sub-clades descending from the *n *clades.

P-values of the homogeneity tests are calculated by Pearson chi-square tests except when only two clades are compared in which case a Fisher exact test is performed, and except when more than two clades are tested and the sample size is less than four in a category. In this latter case p-values are estimated by permutation tests.

The influence of the LD on the power of the different tests is assessed by using the *r*^2 ^measure of LD.

#### Localization of the susceptibility sites

For each replicate, several equiparsimonious trees may exist. We will analyze the *T *first equiparsimonious given by PAUP, *T *being limited to 100 for computation time reasons. For a tree *t*, a new character *S *is allocated to each haplotype *h*. The state of *S *is "0", "1" or "?" depending on the proportion (*p*_*h*_) of cases carrying the haplotype *h *compared to the proportion *p*_0 _of cases in the whole sample.

if , *S *is coded "0" (high number of controls);

if , *S *is coded "1" (high number of cases);

else, *S *is coded "?" (missing data).

with *n*_*h *_being the number of individuals carrying the haplotype *h*.

This character *S *is optimized on the *T *equiparsimonious trees using PAUP and the deltran option. For each site *i*, let  and  be the observed number of times each transition (0→1 for  and 1→0 for ) co-mutates with a 0→1 change of the character *S *on tree *t*. Let  and  be the expected number of co-mutations on tree *t *under the hypothesis of a random distribution of the mutations on tree *t *and an equal probability of mutation on each branch.



where  (resp. ) is the number of 0→1 (resp. 1→0) transitions of the site *i *on tree *t*;

*s*_*t *_is the number of 0→1 transition of the character *S *on tree *t*;

*b*_*t *_is the number of branches of tree *t*.

Then, for each site *i *on tree *t*, we measure the correlated evolution of the site *i *and the character *S*, by defining  (resp. ) as follow:



then, for the *T *equiparcimonious trees, we define  (resp. ) as follow:



Finally, *V*_*i *_is defined as the max between  and . The site or the two sites corresponding to the highest *V*_*i *_are selected as putative susceptibility sites

#### Estimation of power and efficiency

For each of the ten samples of 24 haplotypes simulated along the ten different tree topologies, the process detailed above (attribution of disease status, phylogenetic reconstruction, association test and localization of the susceptibility site) is repeated 1000 times to obtain 1000 replicates, each of them including *N *cases and *N *controls. The power to detect the association is measured by counting the proportion of replicates in which a significant heterogeneity in the distribution of cases and controls is detected between clades. The efficiency of CT to localize the true susceptibility site is simply evaluated as the number of times, among the 1000 simulations, the true susceptibility site is among the detected sites. When two susceptibility sites are simulated, we evaluate the number of times the two sites are detected and the number of times at least one of the two sites is detected.

The test using the cladistic method (CT) is compared to three other association tests: i) a haplotypic test (HT) that compares the haplotype distributions in cases and controls: if *h *different types of haplotypes are observed, a chi-square test with *h *- 1 degree of freedom (df) is performed. As with the CT test, when sample sizes are small, a permutation procedure is used to perform the chi-square test. This HT test is only used to test for association and not to localize the susceptibility sites. ii) An allelic test, referred to as Site by Site Test (SbST), that compares the allele distributions in cases and controls at each site by a chi-square: if the number of sites is *s*, then *s *chi-squares, each with one df, are performed. The site or the two sites that give the most significant results are proposed as susceptibility sites. iii) A test based on CLADHC, the program developed by Durrant et al. [29]. In this test, the data set is analyzed by overlapping windows sliding along the haplotypes. In each window, a tree is reconstructed using a distance method, and a regression analysis is performed at each level of the tree (nested analysis). The level of the tree that maximizes the evidence of disease marker association in the likelihood-ratio test is selected. For each window, the program also provides a significant threshold calculated using the Bonferroni correction. In our simulations, a window size of 6 is selected. We consider the association as detected when at least one of the windows is significant. Concerning the localization of the susceptibility site, CLADHC can only find a window containing the susceptibility site, but not the susceptibility site itself. Therefore, we only use CLADHC in the case of one susceptibility site and we consider the localization as correct when the window with the maximum value of the statistic contains the susceptibility site.

For CT, SbST and CLADHC, we are confronted to a problem of multiple testing. When a real data set is analyzed, a permutation procedure can be used to estimate p-values of the association tests that are corrected for multiple testing. This procedure is very time-consuming and cannot be used on the simulations we have performed here. As an example, the 100,000 repetitions for the Crohn data run in about 24 hours on a Pentium III, 930 MHz, 512 Mo of RAM. Therefore, for SbST and CT, we estimate the nominal type I error required to obtain a global type-one error of 5% by a simulation under the null hypothesis of no association between the studied sites and the disease (5000 replicates). We found that for the different simulation conditions studied here, these nominal errors are equal to 1% for CT and varies from 0.2 to 0.4% for SbST. For the analysis with CLADHC, the program provides a significant threshold calculated using Bonferroni correction. As the Bonferroni correction is very conservative, to obtain similar type I errors with CLADHC as with CT, nominal type I errors were set to values varying from 15 to 17% for simulations with one susceptibility site and from 20 to 25% for simulations with two susceptibility sites.

### Analysis of real data on Crohn disease

The data set is described in a paper of Hugot et al. (2001) [30] and includes 232 nuclear families with two affected children and their parents genotyped for 13 SNPs in the CARD15/NOD2 gene. Haplotypes were reconstructed in these families using GENEHUNTER 2.0b[41]. In each family, one affected child is selected at random to create the case sample (his two haplotypes are labeled cases). The control sample is formed with the parental haplotypes non transmitted to the selected children. The phylogenetic reconstruction assumes that no recombination has occurred between the SNPs. To determine if this was true for the different SNPs tested here or at least for a subset of them, linkage disequilibrium (LD) is estimated between all the SNPs. From the pairwise LD, a group of 7 SNPs (5, 6, 7, 8, 12, 9 and 13) can be identified as forming a block in strong LD among which no or very few recombinations are likely to have occurred (see Additional file 3). Interestingly, this same block of 7 SNPs was described by Vermeire et al. [42] in the CARD15/NOD2 gene. Only these 7 SNPs that form 33 different haplotypes are used in the cladistic analysis. The phylogenetic trees are reconstructed using the same parameters as in the simulations. However here, contrary to the simulations, the ancestral sequence being not known, two different analysis are performed using as ancestral sequence either the most frequently observed haplotype in our sample (mfH) or a consensus sequence formed by the combination of the most frequent alleles at each SNP (consH). For the association test, p-values are estimated on the first equiparsimonious tree obtained by PAUP, using an algorithm similar to the one described in a paper of Becker and Knapp (2004) [43]. This permutation process corrects for multiple testing in a lesser conservative way than the Bonferroni correction because it takes into account the dependency between the tests. For the localization, among all the equiparsimonious trees, 1000 of them are retained and analyzed with CT.

## Authors' contributions

CB perfomed the simulation analyses, contributed to the conception of the study, to the elaboration of the algorithm and wrote the manuscript. VD contributed to the elaboration of the algorithm. J-PH provided the CARD15 data. PD and EG contributed to the design and to the conception of the study and to the manuscript preparation.

## Supplementary Material

Additional File 1 **Average pairwise linkage disequilibrium for the 10 simulated date sets**. The average pairwise LD (over 1000 replicated) is calculated using the *r*^2 ^measure for each of the 10 tree topologies (T1 to T10). The matrices are plotted with GOLD[44]. Click here for file

Additional File 2 Tree topologies of the 10 simulated date sets Click here for file

Additional File 3 **Pairwise linkage disequilibrium for the Crohn data**. (A) shows the pairwise linkage disequilibrium (LD) calculated with the *D*' measure. (B) shows the LD calculated with the *r*^2 ^measures. The matrices are plotted with GOLD [44]. A high degree of LD can be observed. The differences between the *r*^2 ^and the D' values are explained by the difference in the allelic frequency for the different SNPs. Click here for file
