# Supplementary figures and images for: On the use of haplotype phylogeny to detect disease susceptibility loci
Source: BMC Genet. 2005 May 18;6:24. doi: 10.1186/1471-2156-6-24 (PMC1173100; doi:10.1186/1471-2156-6-24)

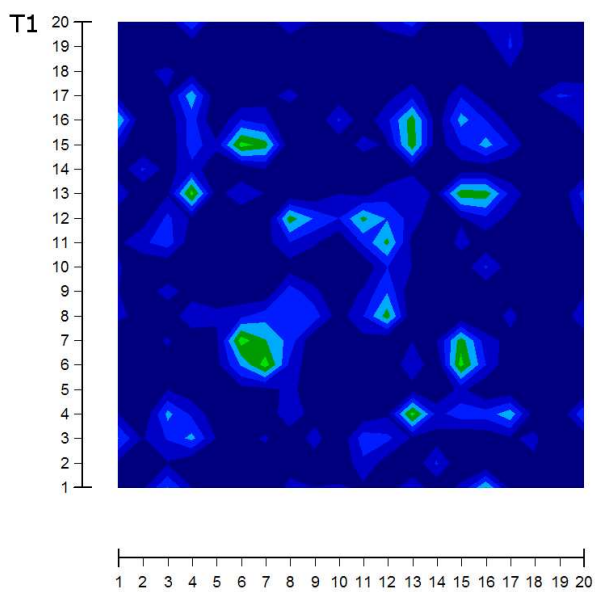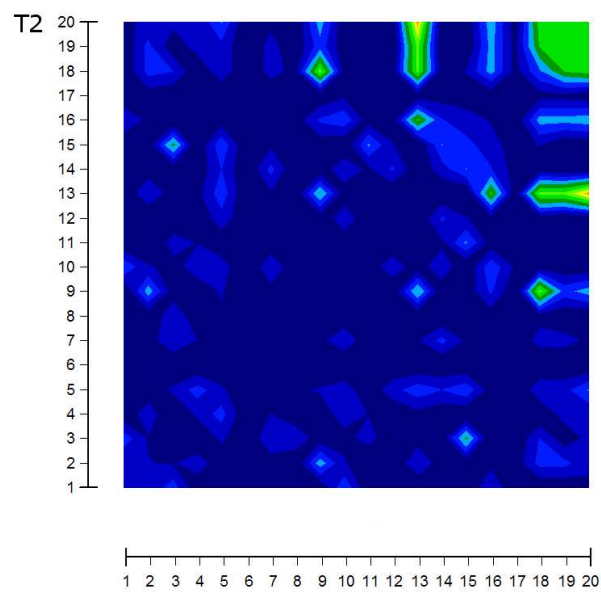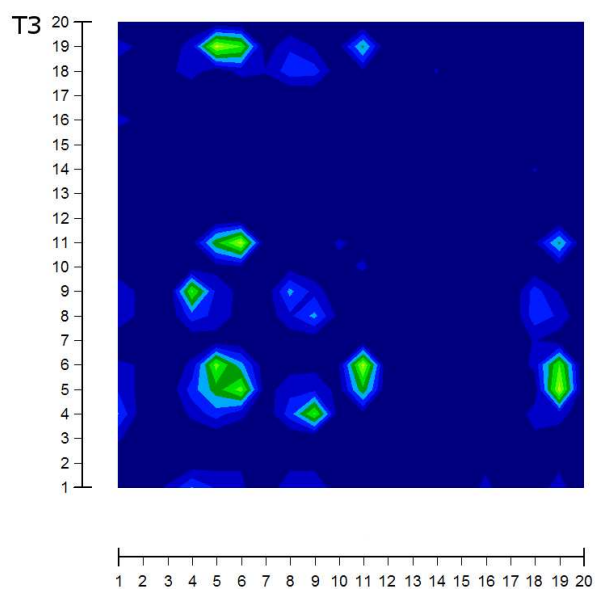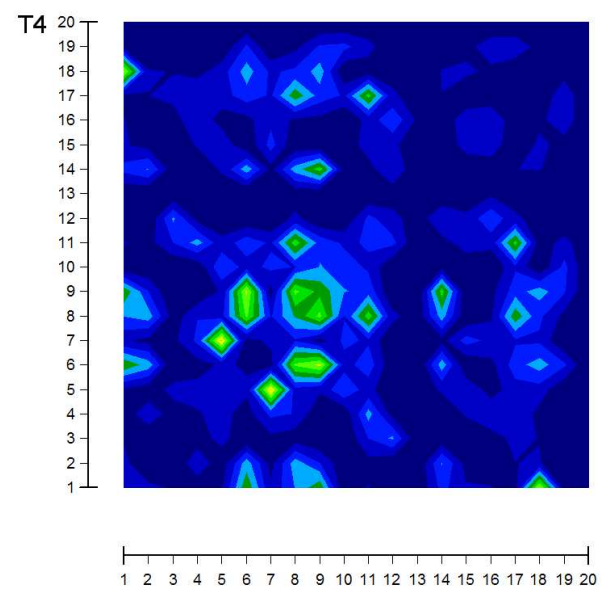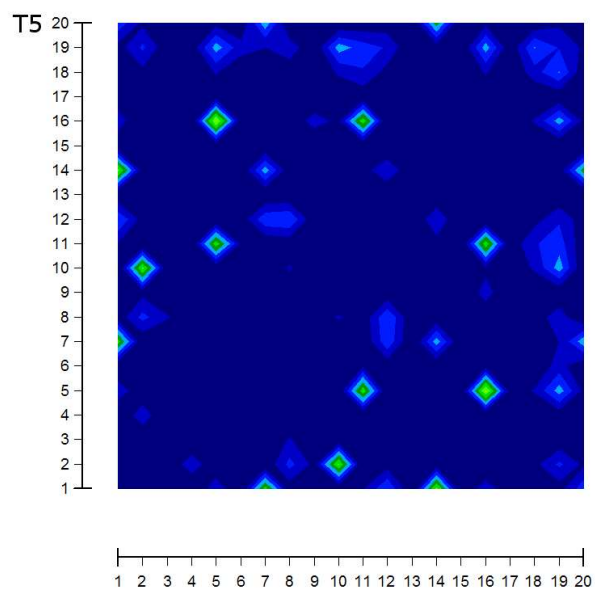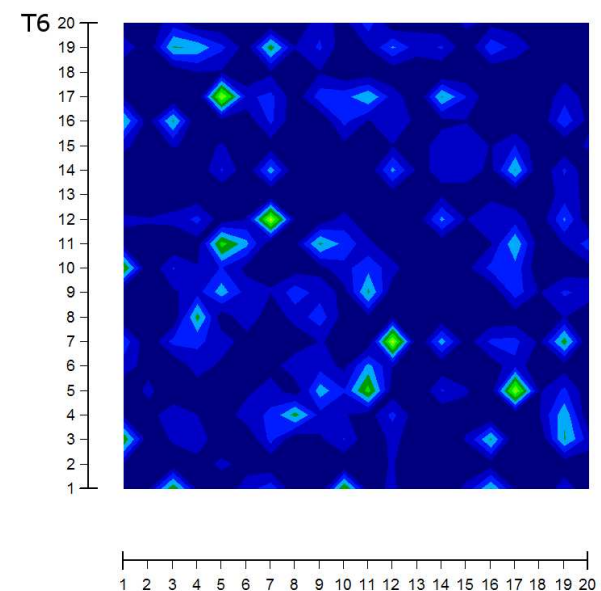

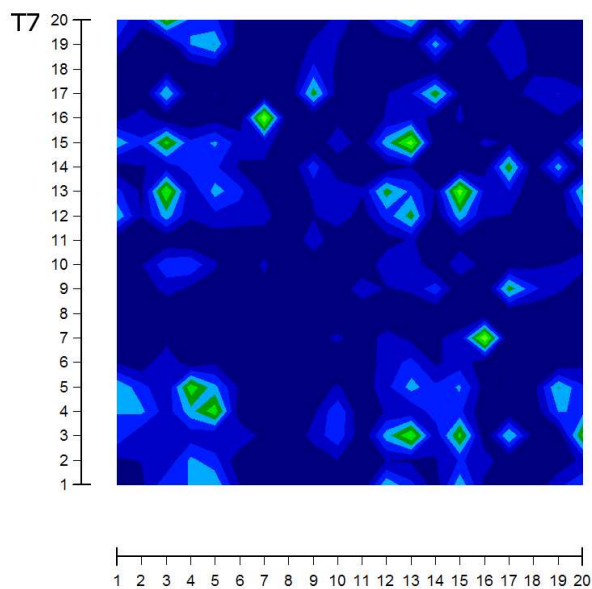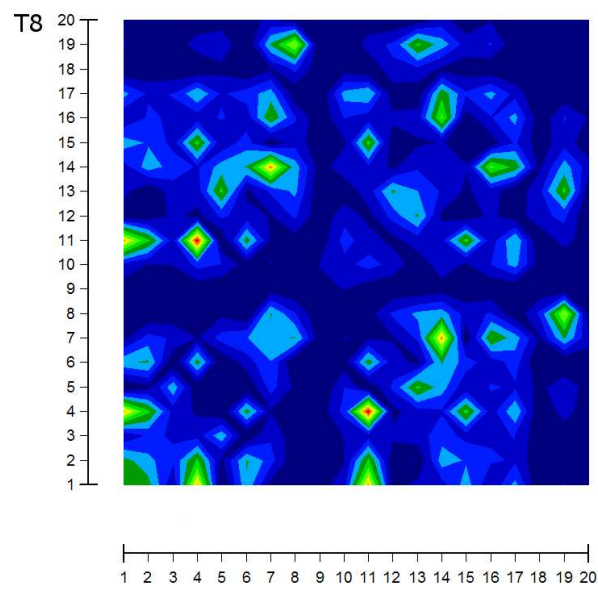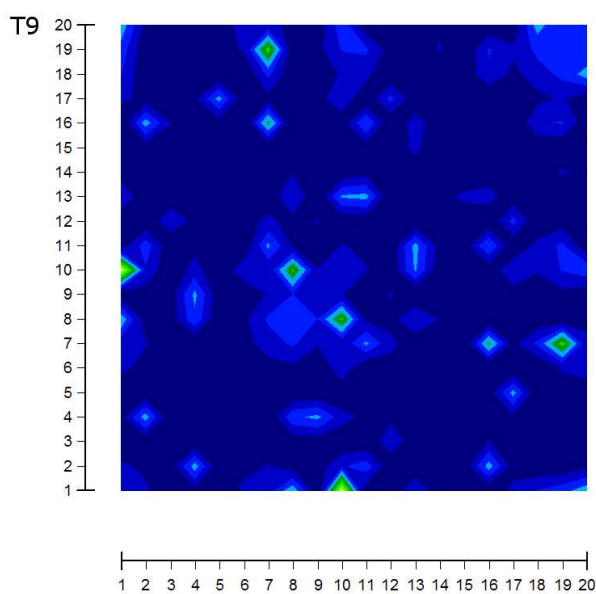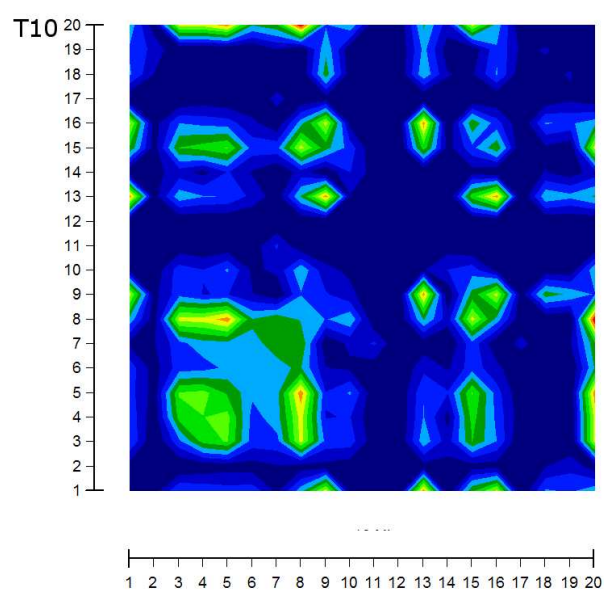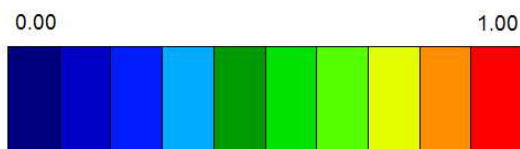

Supplement: Additional File 1 — Average pairwise linkage disequilibrium for the 10 simulated date sets. The average pairwise LD (over 1000 replicated) is calculated using the r2 measure for each of the 10 tree topologies (T1 to T10). The matrices are plotted with GOLD[44]. [file 1471-2156-6-24-S1.pdf]

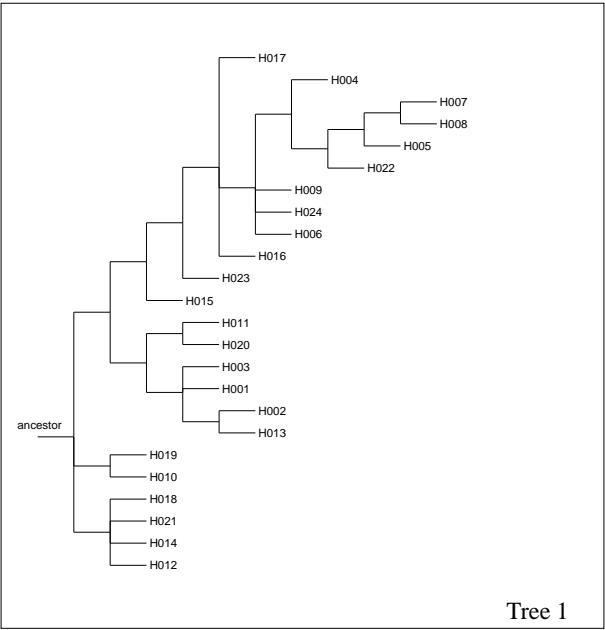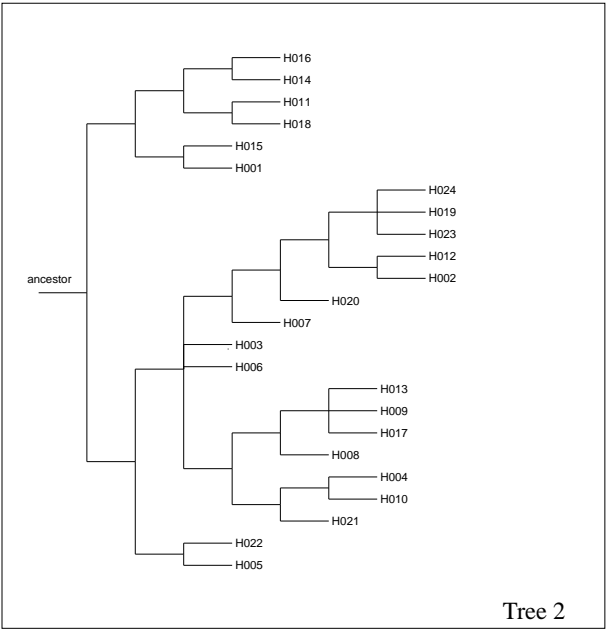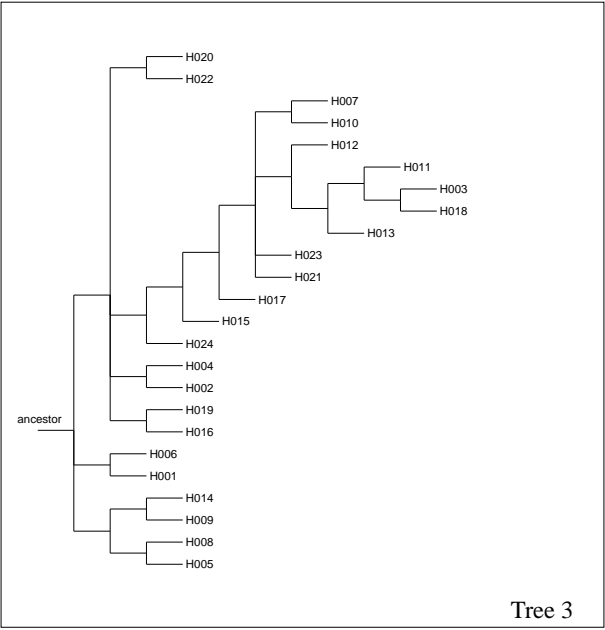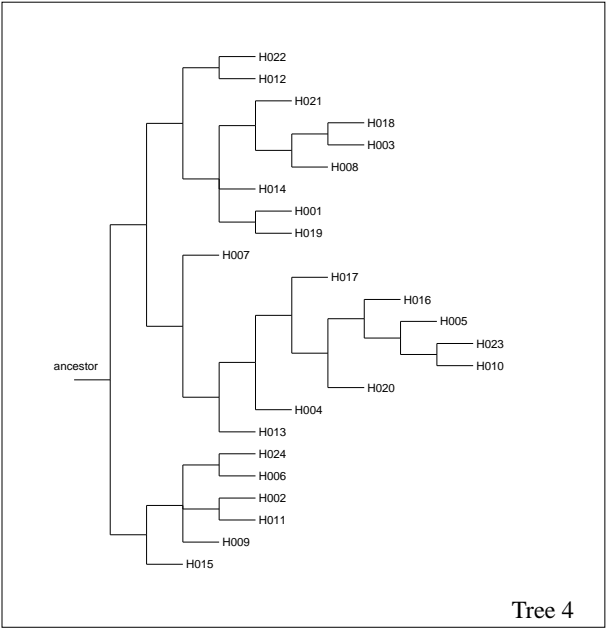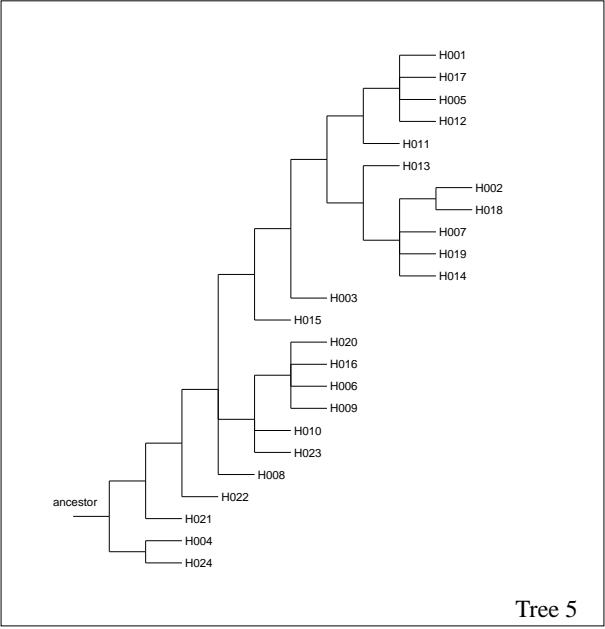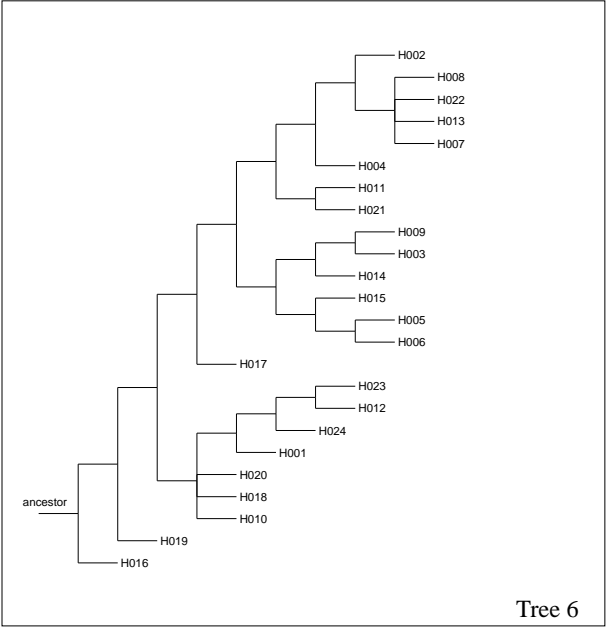

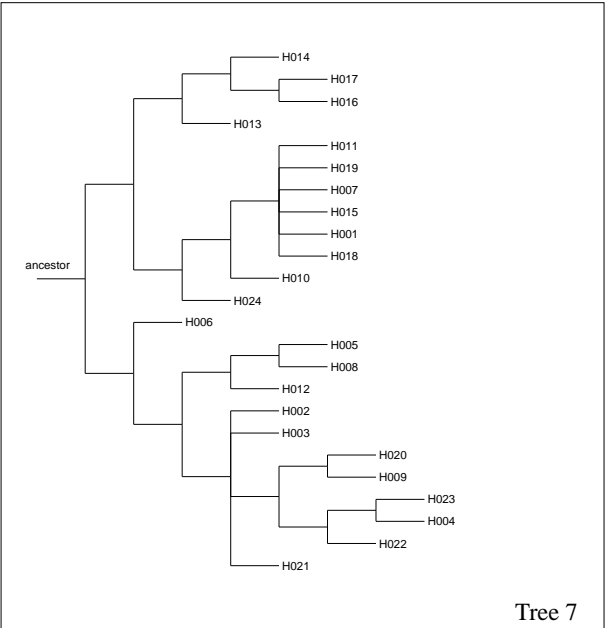

Tree 7

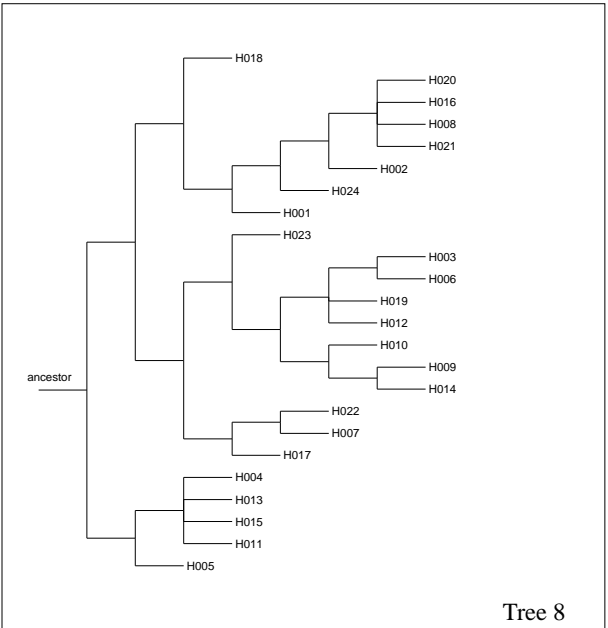

Tree 8

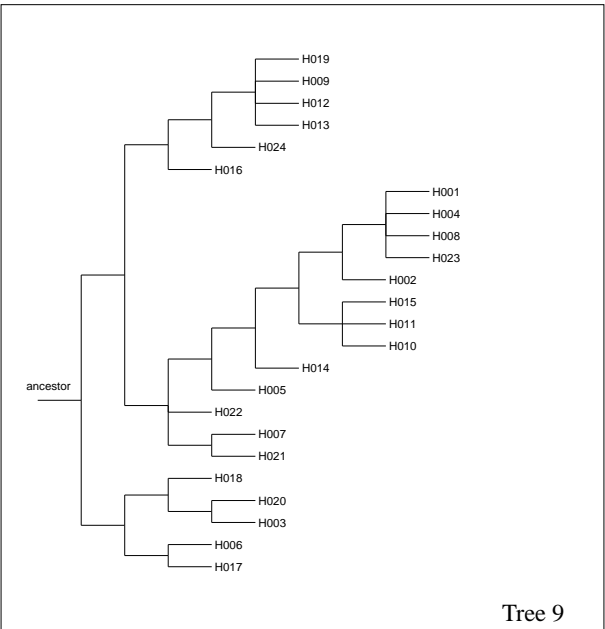

Tree 9

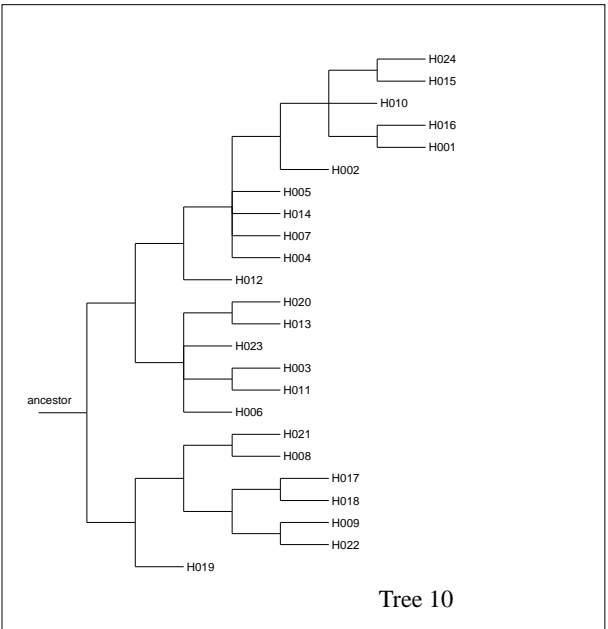

Tree 10

Supplement: Additional File 2 — Tree topologies of the 10 simulated date sets [file 1471-2156-6-24-S2.pdf]

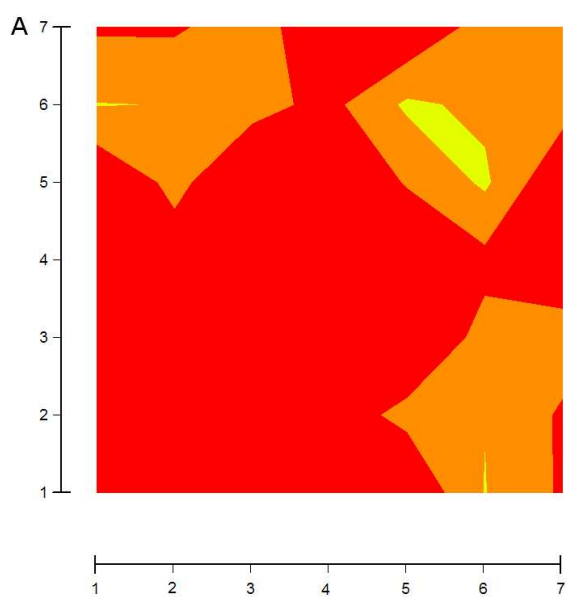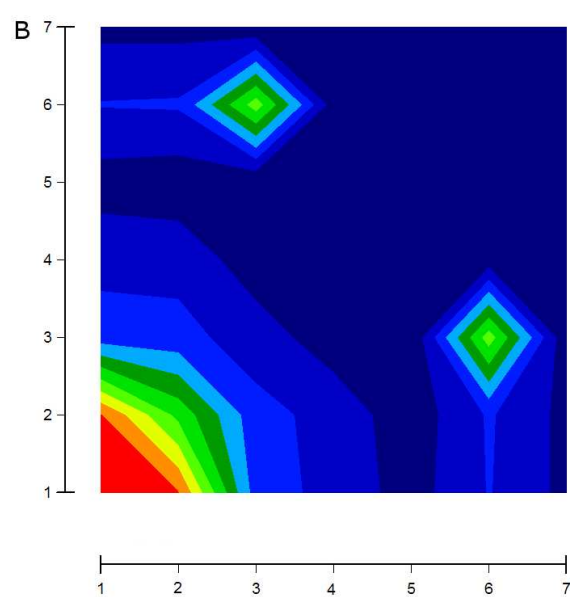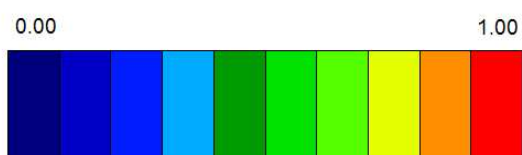

Supplement: Additional File 3 — Pairwise linkage disequilibrium for the Crohn data. (A) shows the pairwise linkage disequilibrium (LD) calculated with the D' measure. (B) shows the LD calculated with the r2 measures. The matrices are plotted with GOLD [44]. A high degree of LD can be observed. The differences between the r2 and the D' values are explained by the difference in the allelic frequency for the different SNPs. [file 1471-2156-6-24-S3.pdf]
